# MRI findings of granulomatous prostatitis induced by intravesical Bacillus Calmette-Guérin treatment for bladder cancer: a comparison with prostate cancer

**DOI:** 10.1007/s11604-025-01871-w

**Published:** 2025-10-10

**Authors:** Hisataka Ito, Takashi Koyama, Shion Tanahara, Daiki Toda, Yoko Akaike, Kenji Notohara, Koji Inoue

**Affiliations:** 1 Department of Diagnostic Radiology, Kurashiki Central Hospital, 1-1-1 Miwa, Kurashiki, Okayama 710-8602 Japan; 2 Department of Diagnostic Imaging and Nuclear Medicine, Graduate School of Medicine, Kyoto University, 54 Shogoin Kawahara-Cho, Sakyo-Ku, Kyoto, 606-8507 Japan; 3 Department of Anatomic Pathology, Kurashiki Central Hospital, 1-1-1 Miwa, Kurashiki, Okayama 710-8602 Japan; 4 Department of Urology, Kurashiki Central Hospital, 1-1-1 Miwa, Kurashiki, Okayama 710-8602 Japan

**Keywords:** Granulomatous prostatitis, Bacillus Calmette-Guérin treatment, Prostate cancer, Magnetic resonance imaging, T1-weighted image, Apparent diffusion coefficient

## Abstract

**Purpose:**

To characterize the magnetic resonance imaging (MRI) features of Bacillus Calmette-Guérin (BCG)-induced granulomatous prostatitis (GP) and to identify key imaging findings for differentiating it from prostate carcinoma (PCa).

**Materials and methods:**

This retrospective study included 11 patients with pathologically confirmed BCG-induced GP and a comparison group of 88 patients (90 lesions) with PCa. Two radiologists retrospectively evaluated MRI findings. Qualitative analysis included lesion location, morphology, and signal intensity (SI) patterns on T1-weighted (T1WI), T2-weighted (T2WI), and diffusion-weighted imaging (DWI). Quantitative analysis compared lesion size, apparent diffusion coefficient (ADC) values, and T1WI SI ratios to both muscle and background prostate. Statistical comparisons were made using Fisher’s exact test for qualitative data and the Mann–Whitney U test for quantitative data.

**Results:**

Hyperintensity on T1WI was significantly more frequent in GP (100%) than in PCa (11%; p < 0.001). The lesion-muscle SI ratio on T1WI was also significantly higher in GP (p < 0.001), whereas the lesion-background SI ratio was not (p = 0.054). Furthermore, a diffuse morphology was significantly more common in GP (45%) compared to PCa (12%; p = 0.014), and the distribution of lesion locations also differed significantly (p = 0.041). No significant differences were found in lesion size, SI on T2WI or DWI and ADC values.

**Conclusion:**

Some PCa exhibit similar imaging findings to GP, but T1 hyperintensity and a diffuse morphology are characteristic features of GP.

## Introduction

Intravesical administration of Bacillus Calmette-Guérin (BCG) is an effective adjunctive therapy for the treatment of non-muscle invasive bladder cancer (NMIBC). Granulomatous prostatitis (GP), a sequela of intravesical BCG therapy, occurs in 41–75% of cases and often clinically mimics prostate carcinoma (PCa) [1]. The increasing use of BCG for NMIBC, along with the increased frequency of transurethral resection of the prostate (TUR-P), contributes to the increasing incidence of GP [1]. While many patients are asymptomatic, others may present with hematuria, fever, obstructive and/or irritative voiding symptoms, and diffuse or focal prostate enlargement on digital rectal examination [1]. Elevated serum prostate-specific antigen (PSA) levels are observed in approximately 75% of patients, and approximately 40% of these cases present with PSA levels of 4 ng/mL or higher. These PSA increases are typically transient, normalizing within 3 months [2, 3]. Definitive diagnosis of GP relies primarily on histopathological examination due to the significant overlap in clinical and imaging findings with PCa.

GP is often asymptomatic, thus initial suspicion of this condition is largely dependent on MRI findings. Elevated PSA levels can also lead to the detection of GP. While a history of intravesical BCG therapy for bladder cancer is an important factor prompting suspicion of GP, differentiation from PCa necessitates a prostate biopsy. Most cases of BCG-induced GP are asymptomatic and do not require intervention. For symptomatic cases, treatment options include fluoroquinolones and antitubercular medications such as isoniazid, rifapentine, pyrazinamide, and ethambutol [4].

Magnetic resonance imaging (MRI) has become a crucial tool in identifying and assessing PCa, primarily utilizing T2-weighted images (T2WI) and diffusion-weighted images (DWI) [5]. PCa typically appears moderately hypointense on T2WI, markedly hyperintense on high b-value DWI, and markedly hypointense on the apparent diffusion coefficient (ADC) map. Previous studies have shown that the MRI characteristics of GP on T2WI and DWI can be similar to those of PCa [6, 7]. As a result, GP is frequently misdiagnosed as PCa, not only clinically but also on MRI. Suzuki et al. reported that diffuse GP lesions typically demonstrate hyperintensity on T1WI and decreased ADC values [6]. However, there are few comparative studies regarding MRI findings in GP and PCa. The purpose of this study was to characterize the MRI features of BCG-induced GP and to examine whether MRI findings on T1WI and ADC values can be useful for differentiating GP from PCa.

## Materials and methods

### Study population

Our Institutional Review Board approved this retrospective study, compliant with the Health Insurance Portability and Accountability Act, and written informed consent was waived. Initially, consecutive patients with BCG-induced GP were identified from our institutional databases. The inclusion criteria were as follows: 1) patients who were pathologically diagnosed with BCG-induced GP based on biopsy, TUR-P, or prostatectomy between June 2011 and September 2023, 2) patients who had a prior history of bladder cancer treated with BCG therapy, and 3) patients who underwent MRI prior to any local or systemic therapy for PCa or GP. Finally, 11 patients with 11 lesions constituted the GP group. For comparison, from the pathological database, consecutive patients pathologically diagnosed with PCa based on biopsy or prostatectomy between February 2022 and January 2023 were included in the PCa group. Patients were excluded if they had: (1) a history of prostate surgery or radiation therapy; (2) lesions not visualized on MRI or incomplete/poor-quality MRI data; or (3) clinically insignificant PCa. Clinically insignificant PCa was defined as ISUP Grade Group 1 (Gleason score 3 + 3 = 6) with a tumor volume < 0.5 mL and confinement to the prostate. The six cases with a Gleason score of 6 in our cohort were included because they were considered clinically significant due to a tumor volume ≥ 0.5 mL. After excluding these cases, 88 patients with 90 lesions constituted the PCa group.

### Clinical data

The medical records of the patients were reviewed for clinical findings. For both the GP and PCa groups, we extracted data on serum PSA levels and the diagnostic procedures used to obtain pathologic specimens. For the GP group, we additionally recorded the intervals between the last BCG therapy session and MRI, as well as the reasons for the MRI examination. For the PCa group, Gleason scores were also recorded.

### MRI protocols

Details of the imaging protocol are summarized in Table 1.

**Table 1:**
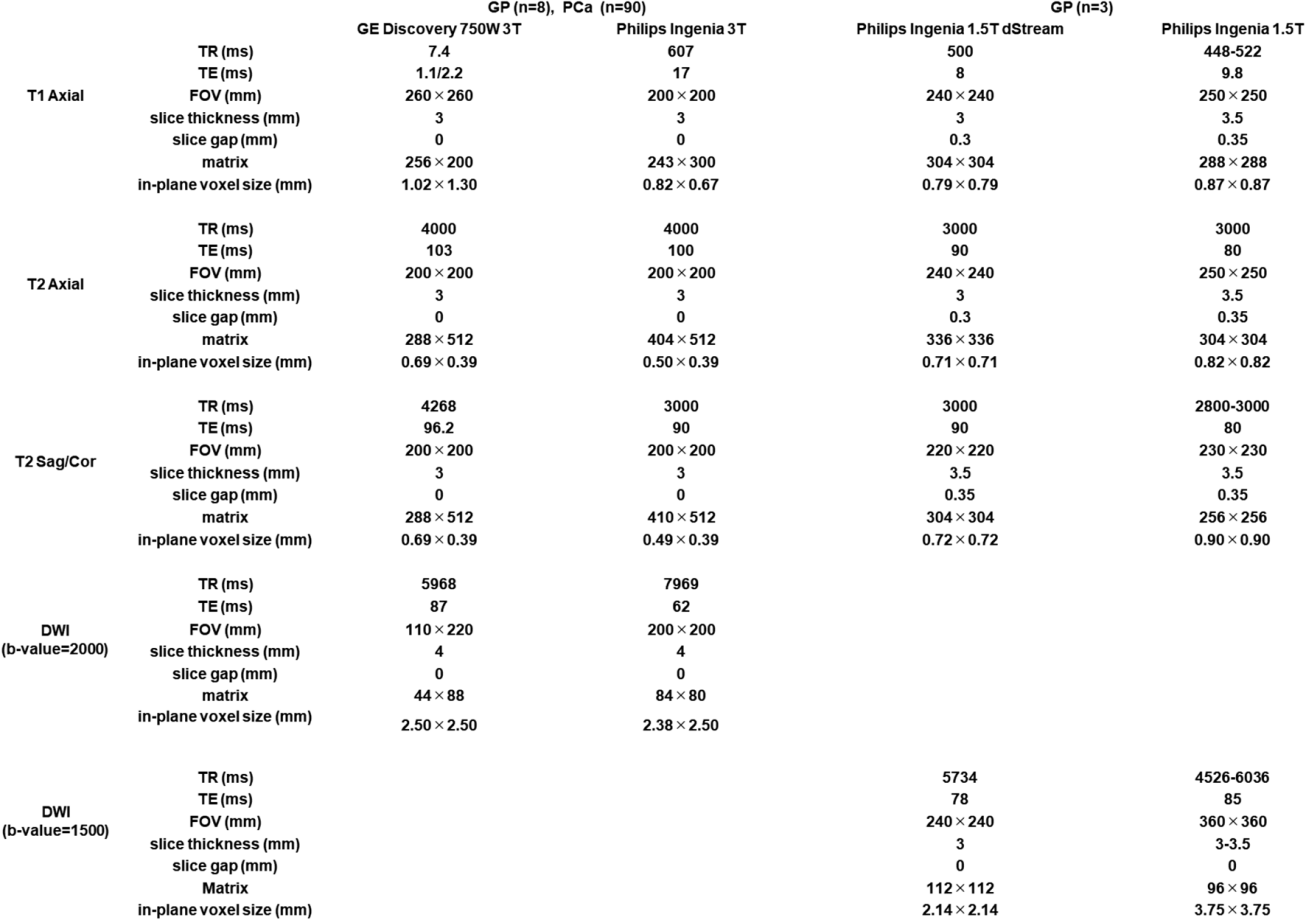
MRI sequence parameters of 1.5T and 3T MRI scanners

*TE e*cho time, *TR r*epetition time, *FOV* field of view, *DWI* diffusion weighted image, *Sag* sagittal, *Cor* coronal

### GP group

MRI examinations were performed using 1.5 T (Philips Ingenia dStream or Ingenia: Philips Medical Systems, Best, the Netherlands) or 3 T (Philips Ingenia: Philips Medical Systems, Best, the Netherlands; or GE Discovery 750w: GE, Milwaukee, WI, USA) MRI scanners. For GP cases, eight cases were imaged using a 3 T MRI scanner, and three were imaged using a 1.5 T MRI scanner. All exams included axial fast spin-echo (FSE) or gradient echo (GRE) T1WI (7.4–607 ms repetition time (TR); 1.1–17 ms echo time (TE); 20–26 cm field of view (FOV); 3 or 3.5 mm slice thickness; 0 or 0.35 mm slice gap; 243 × 304–404 × 512 matrix), axial and coronal or sagittal fast spin-echo T2WI (TR/TE, 2800–4268 ms/80–103 ms; FOV, 20–25 cm; 3 or 3.4 mm slice thickness; 0–0.35 mm gap; matrix, 256 × 256–410 × 512), and axial single shot echo-planar imaging DWI (TR/TE, 4526–7969 ms; 62–87 ms; FOV, 22–36 cm; 3–4 mm slice thickness; 0 mm gap, matrix, 44 × 88–112 × 112; b factors of 0, 1000 and 1500 or 2000 s/mm^2^. In all cases, ADC maps were generated using b-values of 0 and 1000 s/mm^2^. Signal evaluation of DWI was performed using a b-value of 1500 s/mm^2^ on 1.5 T MRI, and a b-value of 2000 s/mm^2^ on 3 T MRI, respectively.

### PCa group

In the PCa group, all cases were imaged using the same 3 T MRI scanner as the GP group.

### Image analysis

Two radiologists with seven and 30 years of experience in pelvic MR imaging, respectively, evaluated all MRI findings in consensus while blinded to the clinical information and pathology results. Radiologic-pathologic correlation was established for each lesion.

Although MRI-targeted biopsy was not performed in all cases, for patients who underwent systematic biopsy, the location of the positive biopsy cores from the pathology report was carefully matched to the location of the lesion identified on MRI by the reviewing radiologists.

 (peripheral zone (PZ) including central zone and/or transitional zone (TZ)) was evaluated on T2WI. Size (maximal diameter in the axial plane) and morphology (diffuse or nodular) were evaluated using T2WI and/or DWI. Well-circumscribed lesions were defined as nodular, while those exhibiting ill-defined margins were categorized as diffuse. Then, the qualitative signal intensity (SI) of the GP and PCa lesions on T1WI, T2WI, and DWI was evaluated. SI was evaluated as follows: on T1WI, SI was compared with that of the obturator muscle; on T2WI and DWI, SI was compared with that of the residual normal PZ. On DWI, evaluation was performed using high b-value images (b = 1500 or 2000s/mm^2^) to avoid T2 shine-through effects.

For quantitative analysis, the two radiologists placed the largest possible ROIs to record the SI from three areas: the lesion itself, the obturator muscle at the same slice level (avoiding visible intramuscular fat), and the surrounding normal-appearing prostate tissue. Using these mean SI values, both the lesion-muscle and lesion-background prostate SI ratios were calculated for all GP and PCa lesions. The two radiologists also recorded the ADC values. To calculate mean ADC values, the largest possible ROI was placed on each lesion. In cases with multiple lesions, these parameters were measured for each lesion separately.

### Statistical analysis

The lesion size, prevalence of hyperintensity on T1WI, lesion-muscle SI ratios on T1WI, and ADC values were compared between the GP and PCa groups. Fisher’s exact test was used to compare categorical variables, including lesion morphology, lesion location, the prevalence of hyperintensity on T1WI and the SI patterns on T2WI and DWI. The Mann–Whitney *U* test was used to compare the lesion size, lesion-muscle and lesion-background SI ratios on T1WI, and ADC values. A *p*-value of less than 0.05 was considered statistically significant. All *p*-values were two-sided. The software used for statistical analysis was R (version 4.5.1, R Foundation for Statistical Computing, Vienna, Austria).

## Results

### GP group

**Fig. 1:**
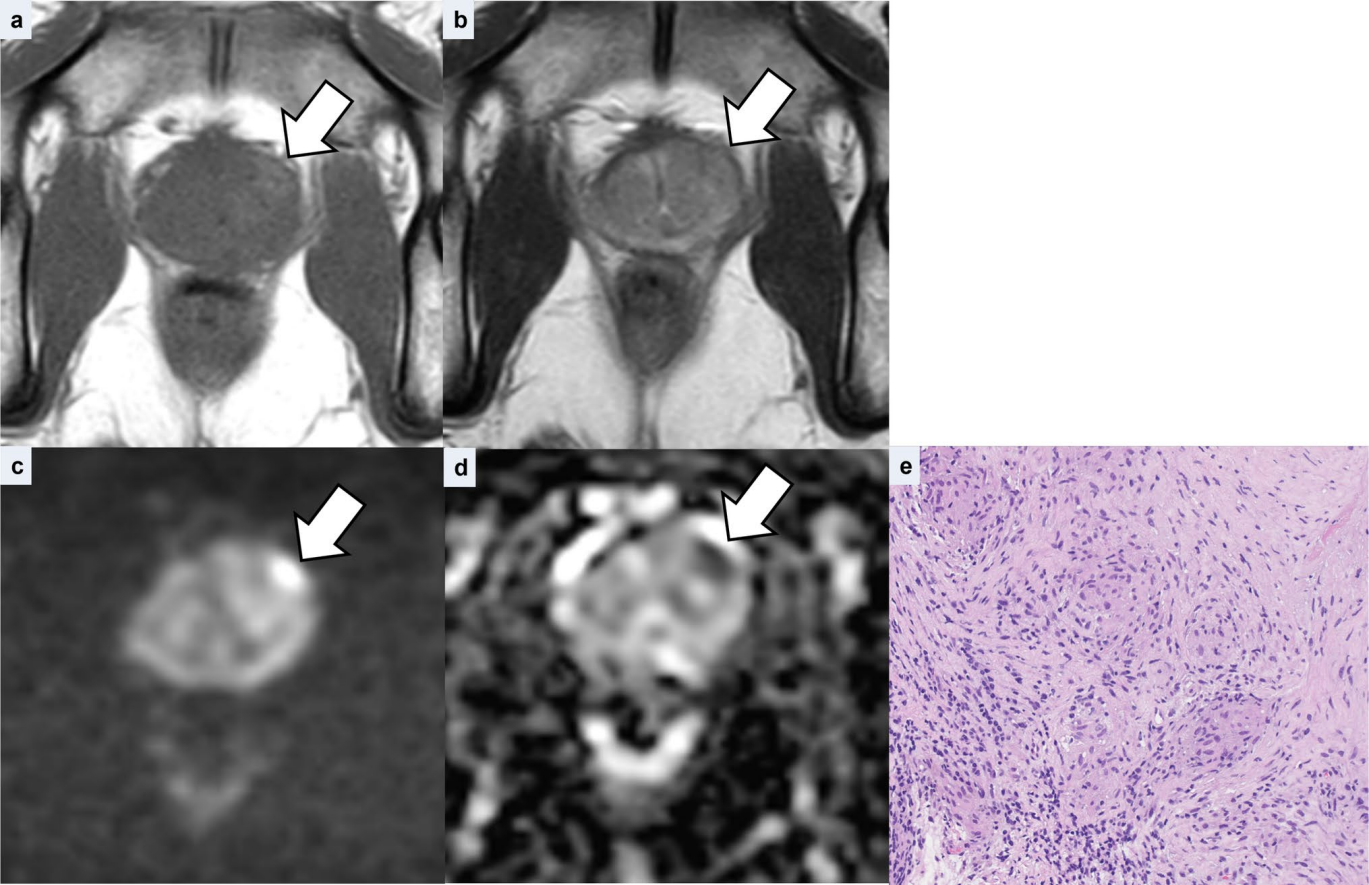
Representative case of nodular lesion of granulomatous prostatitis in a man in his 70s. **a** Axial T1-weighted image shows a hyperintense nodule in the left peripheral zone (arrow). The lesion-muscle signal intensity ratio was 1.40. **b** On the corresponding axial T2-weighted image, the nodular lesion is moderately hypointense (arrow). **c** The lesion exhibits marked hyperintensity on axial diffusion-weighted image with a *b*-value of 2000 s/mm^2^ (arrow). **d** The corresponding apparent diffusion coefficient map shows decreased apparent diffusion coefficient values (0.58 × 10⁻^3^ mm^2^/s) (arrow). **e** Microscopic image shows granulomatous lesions composed of multinucleated giant cells and epithelioid cells without necrosis (Hematoxylin–eosin stain)

**Fig. 2:**
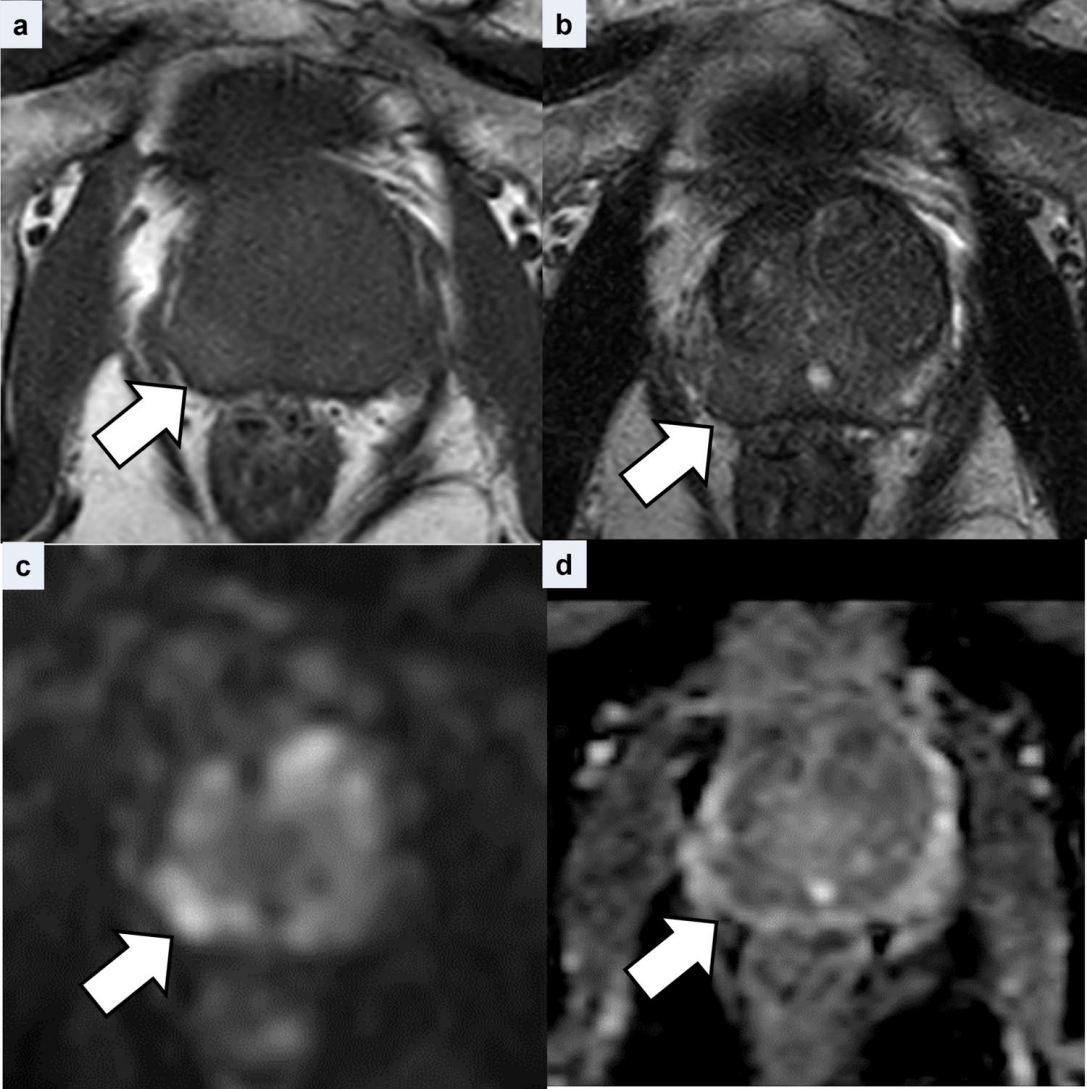
Representative case of diffuse lesion of granulomatous prostatitis in a man in his 70s. **a** Axial T1-weighted image shows diffuse hyperintensity in the right posterolateral peripheral zone (arrow). Lesion-muscle signal intensity ratio was 1.48. **b** On the corresponding T2-weighted image, the lesion demonstrates diffuse, moderate hypointensity (arrow). **c** The diffuse lesion demonstrates hyperintensity on axial diffusion-weighted image (arrow). **d** The corresponding ADC map shows decreased apparent diffusion coefficient values (0.73 × 10⁻^3^ mm^2^/s) (arrow)

The clinical features are summarized in Table 2. Pathologic specimens were obtained by biopsy in ten cases and radical prostatectomy in one case. The case involving radical prostatectomy was performed as part of a radical cystoprostatectomy for BCG-refractory bladder cancer. The GP lesions were solitary in all cases. On pathological review, no incidental coexisting prostate cancer was identified in any of the 11 GP cases. Lesion size ranged from 10 to 25 mm (mean, 15 mm). All lesions were located in the PZ; in four cases, the lesions extended into the TZ (36%). Six lesions (55%) were nodular (Fig. 1) and five lesions (45%) were diffuse (Fig. 2). On T1WI, all lesions showed hyperintensity (100%), and lesion-muscle SI ratios were 1.41 ± 0.19. On T2WI, four cases exhibited hypointensity (36%) and seven cases exhibited moderate hypointensity (64%). Of the four cases demonstrating hypointensity, three were nodular lesions and one was a diffuse lesion. Of the seven cases demonstrating moderate hypointensity, three were nodular lesions and four were diffuse lesions. On DWI, eight lesions exhibited marked hyperintensity (73%), two showed hyperintensity (18%), and one nodular lesion showed isointensity (no abnormality) (9%). ADC values ranged from 0.58 × 10⁻^3^ to 1.6 × 10⁻^3^ mm^2^/s (mean, 0.86 × 10⁻^3^ mm^2^/s).

**Table 2:**
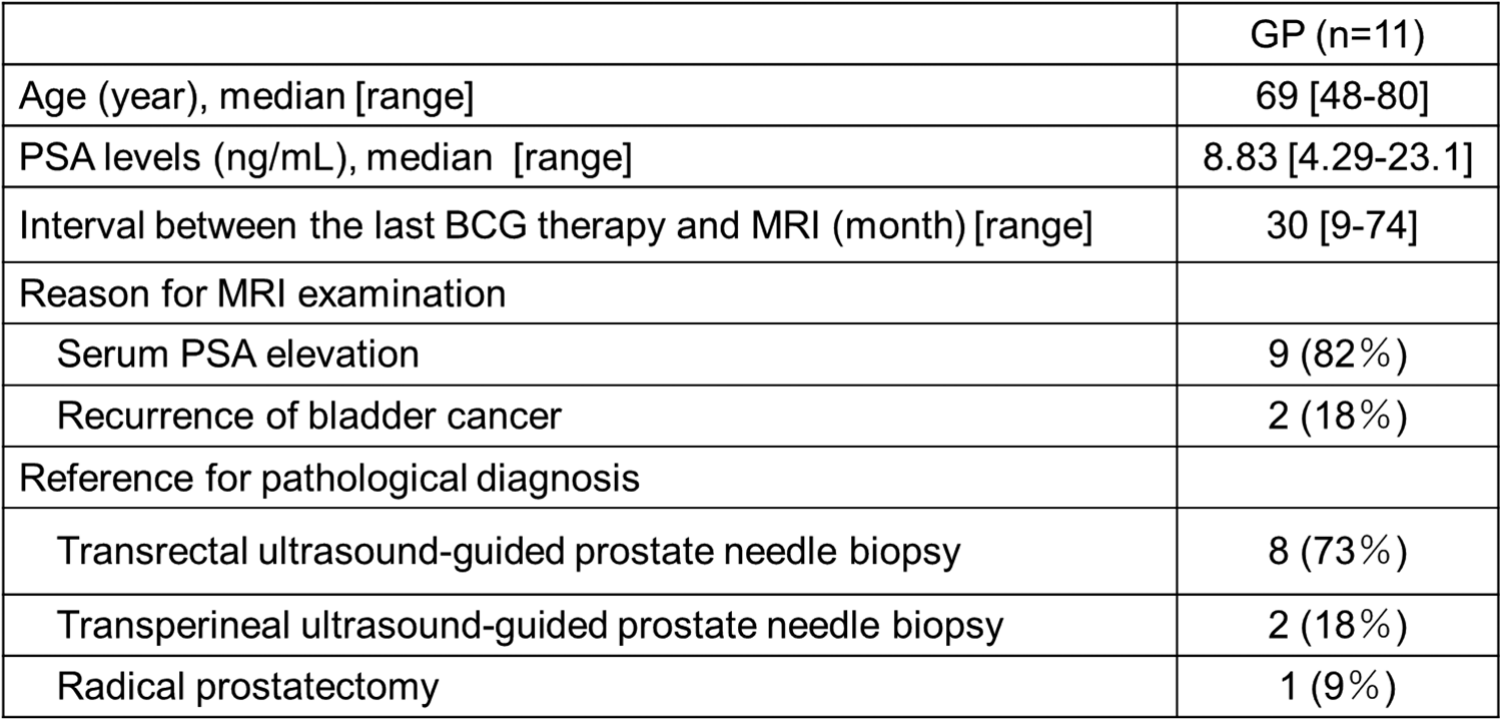
Patient characteristics of GP

*PSA* prostate-specific antigen, *BCG* Bacillus Calmette-Guérin

### PCa group

**Fig. 3:**
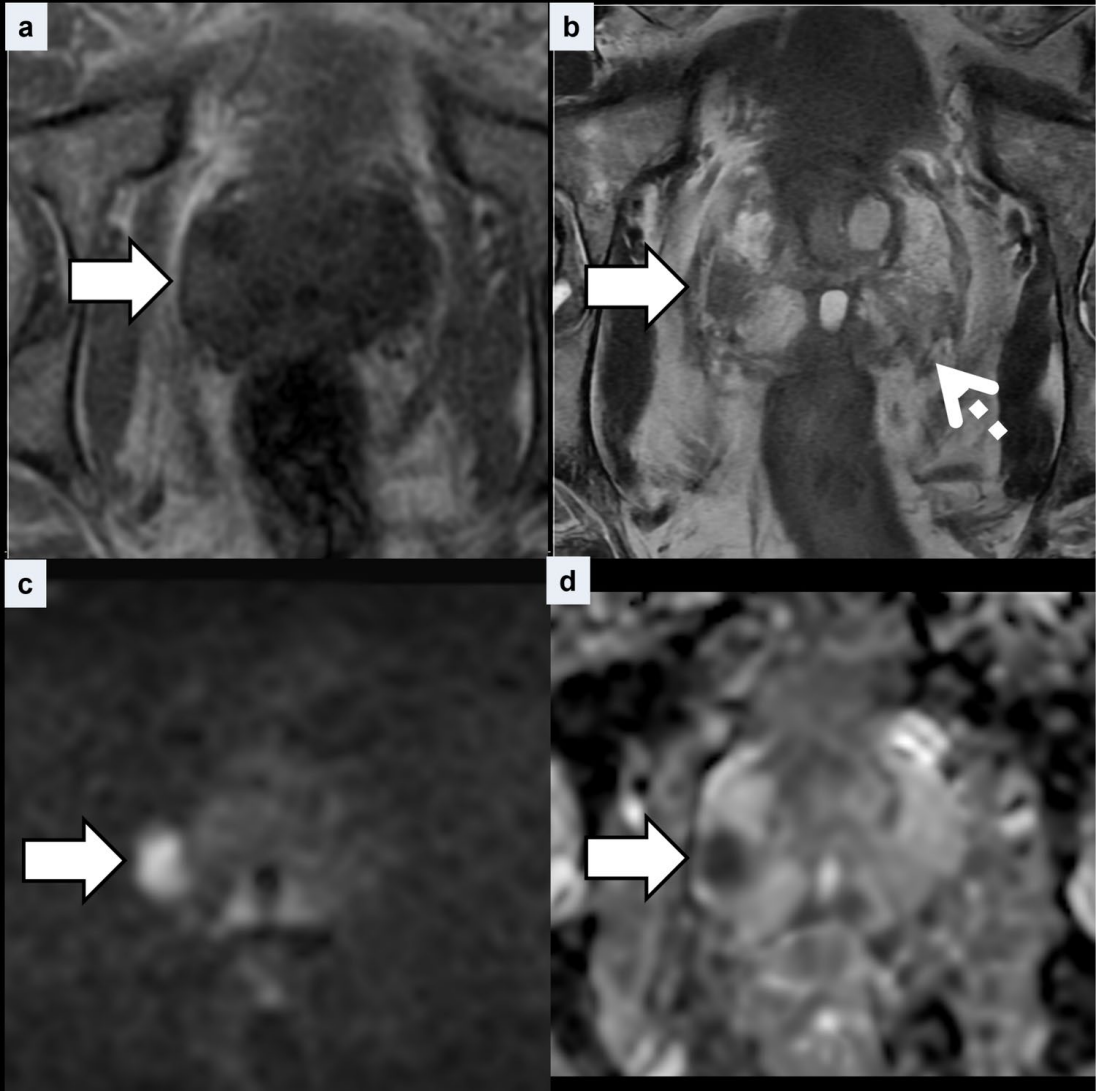
Representative case of prostate carcinoma in a man in his 70s. **a** Axial T1-weighted image shows a hyperintense nodule in the right peripheral zone (arrow). The lesion-muscle signal intensity ratio, measured using a region of interest on the left obturator muscle, was 1.28. **b** On the corresponding axial T2-weighted image, the nodular lesion demonstrates hypointensity with a distinct margin (arrow). A wedge-shaped lesion of moderate hypointensity in the left PZ is consistent with chronic prostatitis (dashed arrow). **c** The lesion demonstrates marked hyperintensity on an axial diffusion-weighted image (arrow). **d** The corresponding ADC map shows decreased ADC values (0.70 × 10⁻^3^ mm^2^/s)

The clinical features and imaging findings are summarized in Table 3. In the PCa group, two patients had two lesions each, while the remaining 86 patients had a solitary lesion. Lesion size ranged from 3 to 54 mm (mean, 16 mm). Hyperintensity on T1WI was observed in 11% of the cases (Fig. 3). PCa-muscle and -background SI ratios on T1WI were 1.14 ± 0.15 and 1.15 ± 0.17, respectively. On T2WI, 69 cases exhibited moderate hypointensity (77%) and 21 cases exhibited hypointensity (23%). On DWI, 71 cases exhibited marked hyperintensity (79%) and 19 cases exhibited hyperintensity (21%). ADC values ranged from 0.35 × 10⁻^3^ to 1.1 × 10⁻^3^ mm^2^/s (mean, 0.79 × 10⁻^3^ mm^2^/s).

**Table 3:**
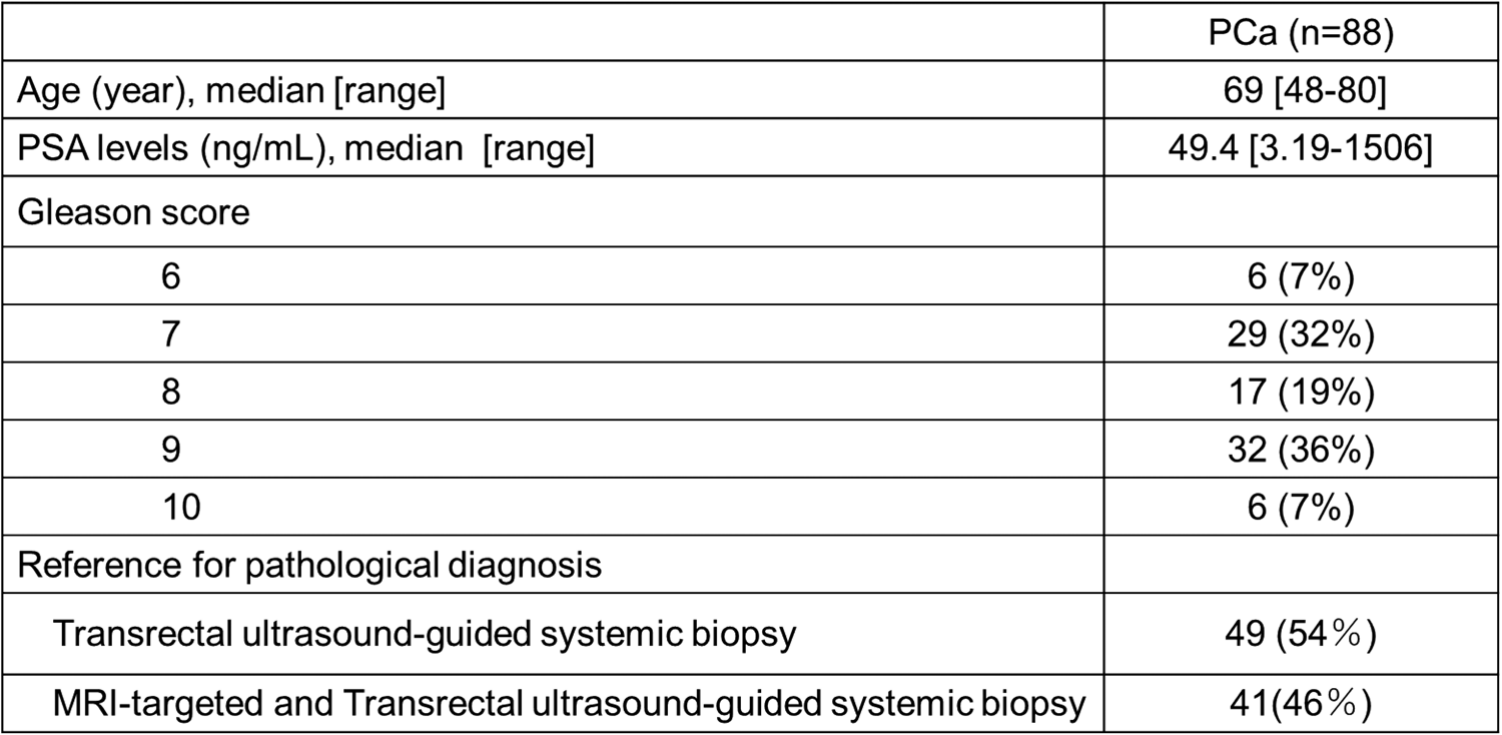
Patient characteristics of PCa

*PSA* prostate-specific antigen

### Comparison between GP and PCa groups

A comparison of the MRI findings is presented in Table 4. Hyperintensity on T1WI was more frequent in the GP group than in the PCa group (p < 0.001), and the lesion-muscle SI ratio on T1WI was significantly higher (p < 0.001). Additionally, significant differences were observed in lesion morphology (p = 0.014) and the distribution of lesion locations (p = 0.041). Conversely, no significant differences were found between the groups in lesion size (p = 0.315), ADC values (p = 0.955), lesion-background SI ratios (p = 0.054), or the distribution of SI on T2WI (p = 0.458) and DWI (p = 0.118).

**Table 4:**
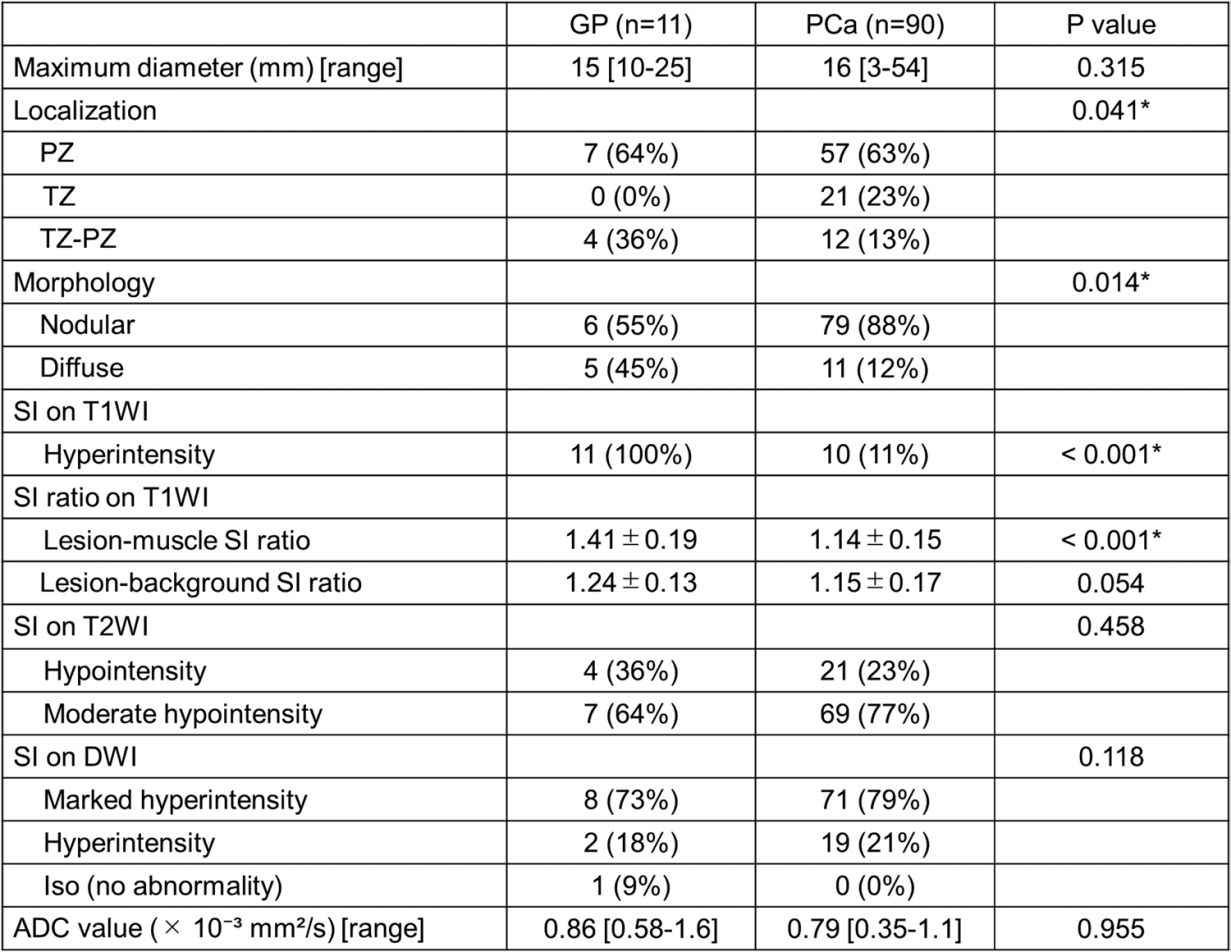
Imaging findings of GP and PCa lesions

*GP* granulomatous prostatitis, *PCa* prostate carcinoma, *PZ* peripheral zone, *TZ* transitional zone, *SI* signal intensity

*Significant difference was observed between GP and PCa (p < 0.05)

## Discussion

Our study showed that MRI in BCG therapy-induced GP is characterized by either nodular or diffuse lesions in the PZ that typically show hyperintensity on T1WI, moderate hypointensity on T2WI, and restricted diffusion. The cause of BCG-related GP is considered to be the intra-prostatic reflux of BCG-contaminated urine. It typically affects the PZ due to the obtuse angle between the PZ and urethra, radiating in a wedge-shaped pattern from the prostatic urethra toward the gland periphery along one or more duct systems [8–10].

In our series, hyperintensity on T1WI was seen in all lesions of GP, regardless of the shape. Our findings suggest that hyperintensity on T1WI might be a more frequent feature of GP than previously reported. The comparison of the prevalence of hyperintensity on T1WI revealed statistical significance between GP and PCa (p < 0.001).

An interesting finding of our study was that the lesion-muscle SI ratios showed a significant difference between the groups, whereas the lesion- background SI ratios did not. One possible explanation for this discrepancy is that the background prostate tissue is a less stable reference than skeletal muscle due to its inherent signal variability. This variability is likely multifactorial. First, as this was a retrospective study, the heterogeneity in MRI acquisition parameters contributed to this variance; specifically, the inclusion of T1WI acquired with both FSE and GRE sequences. Second, the background prostate signal can be intrinsically altered by common coexisting conditions such as chronic prostatitis and fibrosis. In contrast, muscle is less affected by these local pathologies, providing a more consistent signal reference. This suggests that for quantitative T1W signal analysis, using an internal obturator muscle as a reference may be more robust than using background prostate tissue.

Increased SI on T1WI can be attributed to several pathological states. Macrophages engaged in phagocytosis within the granuloma produce paramagnetic free radicals, contributing to both T1 and T2 shortening [11]. In addition, granulomas consist of concentric rings of specialized macrophages surrounding a lipid-rich necrotic core [12, 13]. Within these granulomas, macrophages fuse into multinucleated giant cells, accumulate lipids, and transform into foam cells [14, 15]. These lipids within granulomas also contribute to hyperintensity on T1WI.

Our study has also demonstrated that hyperintensity on T1WI can be seen in 11% of cases of PCa. The hyperintensity on T1WI could potentially be attributed to tumor secretions, but this remains speculative, as a detailed histopathological correlation was not performed and a literature search revealed no prior reports supporting this hypothesis.

The interpretation of T1 hyperintensity in this study requires caution. Our T1WI protocols included two different sequence types: FSE and GRE. While FSE sequences tend to reflect true tissue T1 values relatively faithfully, GRE sequences are highly sensitive to susceptibility effects. Granulomas may contain paramagnetic free radicals produced by macrophages or microscopic hemorrhage, and the GRE technique could have amplified the susceptibility effects from these components, potentially depicting a different SI compared to FSE sequences. Therefore, we cannot definitively conclude that the observed T1 hyperintensity purely reflects tissue T1 shortening, and the variation in imaging sequences may have influenced our signal intensity assessment.

Another key differentiating feature was lesion morphology. In our study, a diffuse pattern was significantly more common in the GP group compared to the PCa group (p = 0.014). While the majority of PCa lesions were nodular, the presence of a non-nodular, infiltrative appearance in a patient with a history of BCG therapy should raise strong suspicion for GP. Additionally, the distribution of lesion locations was significantly different (p = 0.041), primarily because PCa was found within the TZ in some cases, whereas no GP lesions were found exclusively within the TZ in our cohort.

Our study found that the SI patterns on T2WI (p = 0.458) and DWI (p = 0.118) did not differ significantly between GP and PCa. Similarly, regarding ADC values, while several reports have shown that the median ADC value of GP with non-necrotic foci was lower than that of high-grade PCa, our study showed no significant differences between these conditions (p = 0.955) [8, 16]. This considerable overlap highlights the diagnostic challenge and reinforces the need to rely on more specific features like T1WI signal and lesion morphology.

The present study has several limitations. First, the number of GP cases was small. Second, for cases confirmed by systematic biopsy rather than targeted biopsy, the lesion-level radiologic-pathologic correlation is not always precise. Third, as our qualitative image evaluation was based on a consensus method, we did not perform independent reads to calculate inter-reader reliability metrics, such as a kappa coefficient. A fourth and significant limitation of this study was the heterogeneity in T1WI acquisition. Specifically, our protocols included both FSE and GRE sequences, which have substantially different signal characteristics. As T1 SI was the primary endpoint of this study, this difference in sequence type could have influenced both our qualitative and quantitative signal assessments, warranting caution in the interpretation of our findings. Furthermore, although the utility of contrast-enhanced dynamic MRI for evaluating GP has been previously reported, contrast-enhanced MRI was not performed in our study [7, 17]. Variable MRI protocols were used since this study was retrospective in nature. Finally, our study included only unifocal GP lesions, which may represent a selection bias, as we cannot exclude the presence of additional microscopic disease that was undetectable on MRI, particularly since most cases were diagnosed by biopsy rather than whole-gland pathology.

## Conclusion

MRI in BCG-induced GP typically shows hyperintensity on T1WI, moderate hypointensity on T2WI, and restricted diffusion in the PZ. T1 hyperintensity and a diffuse morphology are useful imaging features for differentiating GP from PCa, particularly when BCG therapy for bladder carcinoma is considered.
